# Frontiers in sheep reproduction - making use of natural responses to environmental challenges to manage productivity

**DOI:** 10.1590/1984-3143-AR2022-0088

**Published:** 2022-12-05

**Authors:** Graeme Bruce Martin

**Affiliations:** 1 UWA Institute of Agriculture, Crawley, Western Australia, Australia; 2 UWA School of Agriculture and Environment, University of Western Australia, Crawley, Western Australia, Australia

**Keywords:** sheep, nutrition, pheromone, stress

## Abstract

This review addresses advances, directions and opportunities for research on sheep reproduction in the context of the global challenges of food security and climate change, and demand for ‘clean, green and ethical’ (CGE) animal management. The foundation of CGE management is an understanding of the physiological processes through which the reproductive system responds to changes in the animal’s environment. These days, to the main environmental factors (photoperiod, nutrition, pheromones), we need to add stress from extreme weather events. With respect to nutrition in rams, we now have a deeper understanding of the responses of the brain centres that control gonadotrophin secretion (the kisspeptin system). At testis level, we have found that nutrition affects non-coding RNAs in Sertoli cells and germ cells, thus affecting the balance between cell proliferation and apoptosis. This proliferation-apoptosis balance is also affected during prenatal development, when undernutrition or stress in pregnant ewes seems to elicit epigenetic changes in developing gonads that could affect offspring fertility in adult life. With respect to nutrition in ewes, metabolic signals act directly on ovarian follicles, and thus change ovulation rate, but the variety of signals now includes the adipokines. An early concern was that nutritional supplements that increase ovulation rate would also increase embryo mortality but we now know that embryo survival is improved under field conditions. Finally, we had always thought that the efficiency gains from early puberty in lambs could only be achieved by accelerating fat accumulation, but we now know that faster muscle growth will achieve the same goal, offering two advantages in meat production systems. With respect to pheromones (‘ram effect’), we have a deeper understanding of the brain responses (kisspeptin system) but, most importantly, a realization that the response of ewes to the ram signal involves cell division in memory centres. Many opportunities remain.

## Introduction

The future is difficult to predict but we can be very confident about two major challenges as we approach 2050: i) we will need to feed 50% more people, with increasing demand for animal protein, but shrinking resources for production; ii) global heating will affect the productivity of our food systems due to the effects of extreme weather events. For the livestock industries, these issues are being addressed by the *Global Farm Platform* (GFP, 2022), an international network of research institutions. The original GFP workshop (Eisler et al., 2014) outlined six steps to sustainable livestock: i) Reduce the consumption of human food by livestock; ii) Select livestock species and genotypes that are adapted to the local environment; iii) Recognize the relevance of livestock to local culture; iv) Move towards healthier diets for humans; v) Improve animal health, welfare and nutrition; vi) Reduce environmental footprint.

The most recent GFP workshops, in which Brazil has been represented by Alexandre Berndt and Rui Machado (*Embrapa Southeast Livestock*), described a pathway to achieve sustainability, and the genetic targets related to those goals, in the context of global heating, for a diverse variety of production systems (Rivero et al., 2021a, b). The team at the University of Western Australia works primarily in a dryland environment, in which farming systems face increasingly complex risks (temperature, water, feed security) forcing our livestock production systems to become even more versatile (Blache et al., 2017).

With respect to reproduction in sheep, this paper focusses on two of the initial steps to sustainable livestock: v) Improve animal health, welfare and nutrition; vi) Reduce environmental footprint. Both of these steps are also relevant to an increasing international demand for products that come from ‘clean, green and ethical’ (CGE) management systems: reduced dependence on chemicals (C), smaller environmental impact (G), and to improve animal health and welfare (E). We first presented the CGE management concept in Brazil (Porto Seguro) at the *International Congress on Animal Reproduction* (Martin et al., 2004). This review updates that original presentation and offers directions and opportunities for future research on sheep reproduction.

## CGE management of sheep - update

**Figure 1:**
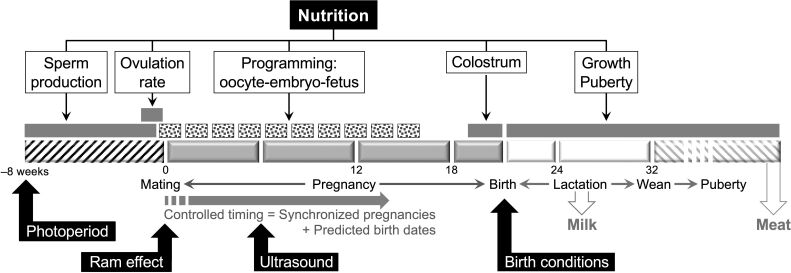
An updated Clean-Green-Ethical strategy for managing reproduction in sheep: periods of ‘focus feeding’ are used to supply timely metabolic signals to the reproductive system to improve outcomes. The nutritional signals can come from short-term supplements or from high-quality pastures. To synchronize the periods of feeding with specific reproductive events, the time of fertilization can be controlled using the ‘ram effect’, or ultrasound can be used to determine pregnancy status and predict time of birth, allowing planning to improve neonatal survival. Modified after Martin (2014) and Delgadillo and Martin (2015).

As can be seen from Figure 1, nutrition remains the central input in CGE management, with outcomes controlled by appropriate metabolic signals supplied with short-term nutritional inputs (‘focus feeding, or ‘nutrient synchrony’ as defined by Hersom, 2008). To ensure that costly supplements are kept to a minimum, it is essential that the supplements are coordinated accurately with events in the reproductive process. For some production systems, the ‘ram effect’ is an obvious tool because it determines the time of conception, but it is limited to some genotypes and to periods when the ewes are anovulatory; for other situations, ultrasound is essential for classifying ewes on the basis of pregnancy status, fetus number and fetus age (Martin, 2014).

Among the obvious changes since our original 2004 presentation are deletion of the risk of nutritional supplements on embryo survival, and an increase in clarity around prenatal ‘programming’, a topic that was speculative but is now supported by solid evidence. These and other advances will be detailed below.

Although the present paper focuses on reproduction, it is important to point out that, in CGE management, a major ‘green’ issue, the mitigation of methane emissions, can actually improve productivity, presenting a ‘win-win’ opportunity, especially if anti-methanogenic forages can be incorporated into the ‘focus feeding’ events suggested in Figure 1 (Blache et al., 2017). In addition, among the ‘ethical’ issues, there is now great interest in neonatal mortality and in stress caused by extreme weather events. The potential role of maternal stress in prenatal development is discussed below. Managing stress is not easy because the animals cannot inform us when they are uncomfortable, although technological solutions might arise from remote sensing of biophysical signals (Maloney et al., 2019). Moreover, genetics can be used to reduce stress while improving meat quality (Zhang et al., 2021) - indeed, genetics can improve almost every aspect of ‘clean, green and ethical’ management in sheep flocks (Martin and Greeff, 2011; Rivero et al., 2021b).

## Updates of aspects of reproduction in the CGE package

a) Brain response to nutrition in the ram

Under normal field conditions, the reproductive capacity of rams can be profoundly changed by both the annual forage cycle and acute nutritional supplementation, with the outcome determined by the latitude of origin of the genotype (Hötzel et al., 2003). For most of our experiments, we used a large (eg, 500 g) supplement of lupin grain as the acute nutritional stimulus because it has high nutritional value but very little soluble carbohydrate and therefore avoids rumen acidosis. In early studies, we explored neuroendocrine and endocrine responses and described: i) the metabolic and endocrine inputs to the brain-pituitary (nutrients, metabolites, substrates, insulin, leptin); ii) the changes in the frequency of GnRH pulses (bioassayed by observing LH pulses), and the secretion of gonadotrophins, inhibin and sex steroids; iii) the changes in the intensity of negative feedback exerted by the sex steroids, either testosterone or the products of its intracerebral aromatization (review: Blache et al., 2003; Martin et al., 2010). More recently, we have shown that the nutritional stimulus interacts with the trio neuropeptides - kisspeptin, dynorphin and neurokinin - the ‘KNDy’ system in the arcuate nucleus (ARC) that controls GnRH pulse frequency. When rams were given a supplement of lupin grain, the increase in LH pulse frequency was associated with activation of kisspeptin neurons in the ARC and the dorsomedial hypothalamus (Rietema et al., 2019).

The interactions that underpin these responses at brain level are complex, necessitating experimental control over genotype, season (photoperiod) and dynamic changes in sex steroid status. When these interactions are superimposed on in-vivo experiments that require serial blood samples (typically every 20 min for 24 h), and a need for replication in the design, the logistical limitations are difficult to overcome. We therefore decided to use a dynamic compartmental model of the ram reproductive axis so we could detect critical control points. The model could emulate the spectrum of endocrine responses that we had observed in several experimental situations, including the responses to acute nutritional supplementation. Most striking was the suggestion that the inhibitory action of testosterone involved a variable time delay, with the model producing single pulses or double pulses, as we had observed during puberty or during nutrition-induced testicular growth. In other words, there is a dynamic interaction between the GnRH pulse generator and negative feedback by gonadal steroids, involving changes in a time delay in feedback that could help explain responses to nutrition, and perhaps photoperiod and socio-sexual signals (Ferasyi et al., 2016). We do not know yet how such a time delay could be implemented, but possibilities include the process of aromatization of testosterone to oestrogen at brain level, or perhaps interactions with the ‘KNDy’ system that controls GnRH pulse frequency.

b) Testis responses to nutrition

Although we have focused a lot of attention on the role of brain-controlled gonadotrophin secretion, there is also strong evidence that a proportion of the testis response is independent of those processes, although we have little understanding of the nature of the ‘GnRH-independent pathway’ (Hötzel et al., 1995). This is a neglected area that requires investigation.

However, we have not completely ignored the target organ, and completed several studies of the effects of nutrition on testis structure and function. It has long been known that changes in nutrition induce reversible changes in testis mass and total sperm production, and that these responses are associated with changes in spermatogenic efficiency, and in spermatozoa quality as determined by motility and DNA damage (review: Guan and Martin, 2017).

It is important to note that we use sexually mature rams. This context is critical because, when the regulation of spermatogenesis is being considered, there is an inevitable focus on Sertoli cells and these cells become terminally differentiated at puberty and lose their ability to divide. Therefore, they are not likely to be a major component of the large gains in testis mass that we can induce. In fact, our early observations challenged this dogma, but later studies with better techniques confirmed that the changes in testis mass in the adult ram are not accompanied by a change in Sertoli cell number, although a few Sertoli cells do appear to retain proliferative ability (review: Guan and Martin, 2017). By contrast, Sertoli cell function was greatly affected. During testis regression induced by underfeeding, tight junctions became disorganized and Sertoli cell differentiation and maturation seemed to be reversed - these outcomes that are coherent with an increase in germ cell apoptosis and a decrease in the rate of spermatogenesis, two major contributors to the reduction in of spermatogenic efficiency. These responses were related to two RNA-based processes: i) the expression of small non-coding RNAs that are involved in the regulation of Sertoli cell function, spermatogenesis and germ cell apoptosis; and ii) alternative pre-mRNA splicing that affects regulation of spermatogenesis but does not appear to affect germ cell apoptosis, at least during testis regression induced by undernutrition. This is a new perspective on how intra-testicular processes, especially those mediated by RNA, affect the fertility of male livestock.

c) Ovary responses to nutrition (ovulation rate)

In the ram, the effects of nutrition on sperm output are initiated by brain responses and appear to be sustained by gonadal processes. In the ewe, however, brain processes determine the ‘decision to reproduce’ (ie, to ovulate) but increases in ovulation rate are not caused by a more intense drive to reproduce. Rather, the effects of nutrition on ovulation rate are mediated by direct metabolic inputs into folliculogenesis. Scaramuzzi et al. (2011) presented a consensus on those processes with an emphasis on nutritional effects on the viability of gonadotrophin-dependent follicles, leading to a greater flow through to gonadotrophin-responsive follicles, and thus more ovulatory follicles.

Recently, in a new consensus paper, Juengel et al. (2021) summarized subsequent advances including the paracrine and autocrine actions of the adipokines (eg, adiponectin, chemerin). This situation is complex because most adipokines are produced in more than one variant and, to date, many variants have not been measured. Moreover, the current experimental approaches are limited because most of our knowledge comes from *in vitro* experiments with bovine tissues, and most studies report the effects of one or two adipokines, thus ignoring the possibility of additive, antagonistic or synergistic effects among adipokines. Clearly, there is a need to consider the full ‘adipokinome’ if we are to understand these new metabolic inputs into folliculogenesis.

d) The ram effect - brain responses to pheromones in the ewe

Pheromones from a novel ram activate neuroendocrine pathways in the ewe, rapidly increasing GnRH-LH pulse frequency (Hawken et al., 2009). There are significant gaps in our understanding of this ‘ram effect’, many of which can be traced back to misconceptions in early documentation or to uncontrolled factors in experimental design, particularly the lack of clarity around the novelty status of the rams used in almost all experiments (review: Jorre de St Jorre et al., 2014). Therefore, the need for novelty in the stimulus rams was a profound discovery because it implied that individual males must emit a specific pheromone and that ewes can distinguish among individual rams and remember them. Inevitably, the next question was: how do ewes form an ‘olfactory memory’? The answer seems to involve neurogenesis in the memory centres of the brain - within 2 h of exposure to novel rams, there a rapid and robust increase in the rate of cell proliferation in the dentate gyrus of the hippocampus (Hawken et al., 2009). It is fascinating that the odour of a new-born lamb induces cell division in the same region of the ewe brain, presumably driving the mother-young recognition that is critical for neonatal survival (eg, Corona et al., 2018). In both situations, little work has been done on the chemical structure of the pheromones involved.

We have investigated the neuronal processes leading to the endocrine response (Hawken et al., 2019). Within 2 h, the ram stimulus activates neurons in the arcuate nucleus (ARC), the ventromedial nucleus of the hypothalamus (VMH), and the organum vasculosum of the lamina terminalis (OVLT). Interestingly, cells in the preoptic area (POA) did not become activated until perhaps 6 h later. We would expect a reversal of all of these outcomes when the ram stimulus is removed, but only the basal and mean LH concentrations decline, and there is no change pulse frequency. Stimulus removal decreases the number of Fos-IR cells in the ARC, VMH and OVLT, but not in the POA. This rather complex pattern of neuronal activation is probably explained by interactions with the two populations of KNDy cells (only one of which controls GnRH pulse frequency), and the effects of changes in circulating oestradiol concentrations on negative and positive feedback (De Bond et al., 2013; Fabre-Nys et al., 2017; Hawken et al., 2019).

e) The ram effect - interactions with nutrition

As a management tool, the ram effect has limitations, including: not all anovulatory ewes respond; many of the ewes that ovulate have a very short luteal phase; and cyclicity often does not persist in successfully stimulated ewes (review: Martin, 2014). Early on, we had shown that the ovulatory response was improved by a long-term improvement in ewe body condition for the 5 months before the ram stimulus was applied (Fisher et al., 1993). We subsequently tested whether any of the limitations to the response could be resolved by offering the ewes a short-term lupin supplement before ram introduction, on the premise that metabolic signals from such supplements activate ovarian follicles (review: Viñoles et al., 2014). The hypothesis was rejected because the only gain was the expected increase in prolificacy (Scaramuzzi et al., 2014; Khaiseb et al., 2022). The underlying mechanisms for all of these issues requires further research.

With respect to the strength of the ram stimulus itself, any factor that improves the expression of pheromonal characteristics would be expected to improve the ovulatory response of ewes. However, Fisher et al. (1994) found that lupin supplementation of Merino rams for 17 weeks increased testis size, as expected, but did not affect the percentage of ewes induced to ovulate, perhaps because this nutritional treatment has little effect on the circulating concentrations of testosterone (Hötzel et al., 1995), an essential factor in pheromone production by male sheep (Fulkerson et al., 1981). Interestingly, in contrast with the sheep, well-fed goat bucks are more effective at inducing ovulation in does than underfed bucks (Walkden-Brown et al., 1993; Delgadillo et al., 2021). It will be difficult to resolve this situation until we have some way to quantify pheromone production.

f) The ram effect - non-olfactory ‘pheromones’?

Recently, Robertson and Martin (2022) made a case for re-defining ‘pheromone’ in the context of chemical communication between the sexes for sheep, as well as pigs, rodents and humans. We proposed expanding the concept from purely olfactory signals to include signals in seminal plasma - it carries strong molecular signals (eg: transforming growth factor-ß; 19-OH prostaglandins; ligands of Toll-like receptor-4; cyclic ADP ribose hydrolase) that act on the female reproductive tract to enhance the prospect of pregnancy. It is important to remember that the genetic makeup of the sperm and the conceptus are different to that of the prospective mother, so her immune system would see these intruders as ‘foreign’ and therefore reject them. By modulating the maternal immune system, the ‘seminal pheromones’ ensure that the uterus is receptive to the embryo and make pregnancy possible. A broader pheromone concept would bring new perspectives to reproductive biology, and should stimulate investigation into the loss of important signaling molecules in artificial insemination programs.

g) Embryo mortality

At the time of the original 2004 presentation, we were concerned that nutritional supplements immediately after fertilization would increase progesterone clearance and thus cause early embryo mortality, thus nullifying any gain in prolificacy. We have now realized that, under field conditions, nutritional supplements can actually reduce embryo mortality, rather than increase it, allaying our fears (Viñoles et al., 2012).

h) ‘Programming’ future reproductive performance - from gamete to embryo to fetus

**Figure 2:**
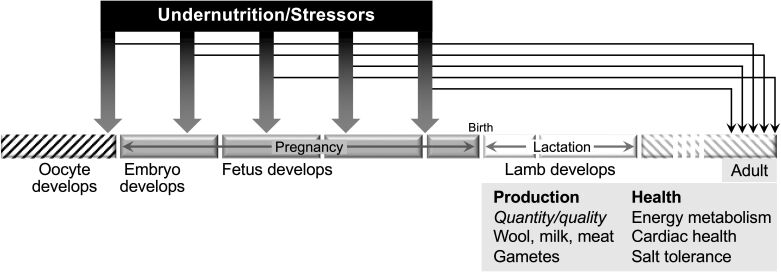
Schematic representation of the ‘Developmental Origins of Health and Disease’ (DoHaD) as applied to reproduction in the sheep. Undernutrition and stress in the pregnant ewe act on the oocyte, embryo or fetus, probably through epigenetic processes, and ‘program’ the health and productivity of the mature offspring.

The concept of ‘Developmental Origins of Health and Disease’ (DoHaD) began with the epidemiology of maternal undernutrition in humans and was then extended to farm animals (review: Sinclair et al., 2016). There is now strong evidence for ‘programming’ of diverse aspects of future health and productivity in sheep (Figure 2) - eg, the quantity and quality of wool, milk and meat produced (eg, Kelly et al., 2006; van der Linden et al., 2009; Sen et al., 2015); cardiovascular health, salt tolerance, thyroid function, and glucose homeostasis (Gopalakrishnan et al., 2004; Chadwick et al., 2009; Jaquiery et al., 2016; Johnsen et al., 2018). Interestingly, the DoHaD hypothesis began with a focus on the fetus but has now been extended back to before fertilization because there is evidence that undernutrition also causes effects in the oocyte and embryo that have consequences for postnatal life (Ashworth et al., 2009; Jaquiery et al., 2012). That said, research is still needed to determine whether such effects have important consequences for reproductive efficiency of mature animals under industry conditions.

Nutritional ‘programming’ of ewe reproduction: the early discoveries of DoHaD in the sheep, including effects on puberty and gonadal development, were reviewed comprehensively by Rhind et al. (2001). Subsequently, Vinoles et al. (2014) took advantage of very long-term studies of Merino ewe nutrition to show that endocrine profiles, follicle development and ovulation rate in adult ewes are affected by the nutrition of their mothers during pregnancy, even after a delay of 3-5 years. These observations are consistent with those by Mossa et al. (2017) who found that nutritional imbalances during fetal life negatively program the size of the ovarian reserve, serum AMH concentrations and potential fertility, in adulthood. Recently, Nwachukwu et al. (2021) suggested that it was maternal dietary protein that regulated ovarian development in the offspring - however, as with all studies in ruminants, there is a risk in such conclusions because the rumen can interconvert energy and protein. There is a lot of scope for further research into the reproductive aspects of DoHaD in the ewe.

Nutritional ‘programming’ of ram reproduction: maternal undernutrition of the ewe during pregnancy can affect the development of the fetal testis, with consequences for the number of Sertoli cells (Alejandro et al., 2002; Rae et al., 2002; Kotsampasi et al., 2009; Hoffman et al., 2018), apparently through processes mediated by intratesticular changes in STAR protein and IGF-I (Mossa et al., 2018). On the other hand, in another study, ewe undernutrition from mating to Day 110 of gestation had no effect on the number of Sertoli cells, or on apoptosis pathways, in the developing testis (Andrade et al., 2013). The inconsistency among reports is perhaps explained by the timing of the nutritional restriction, an issue that requires space more research.

The proliferation of Sertoli cells is an obvious target for DOHAD because Sertoli cell number is fixed at puberty, and adult fertility is largely determined by the ratio of Sertoli cells to germ cells. Indeed, as shown by studies in rats, maternal undernutrition changes the balance between cell proliferation and apoptosis in the developing testes, an essential aspect of the regulation of spermatogenesis (Pedrana et al., 2021).

The role of maternal undernutrition in the development of the reproductive system of ram lambs could explain much of the between-animal variation in twinning and sperm production that typifies sheep flocks on farms. Moreover, it could contribute to between-ram variation, a problem that plagues selection of rams for experimentation and for mating programs. We need to consider the DOHAD history of the animals we use.

DoHaD and maternal stress and in the ram lamb

Stress imposed on the mother during pregnancy might be expected to affect the developing fetus because it increases the maternal secretion of glucocorticoids. This issue has been studied in depth in the ram lamb by Graciela Pedrana in Uruguay. She has shown that the Leydig cells in the ram fetus contain glucocorticoid receptors, and that receptor density is increased by prenatal glucocorticoid treatment (Pedrana et al., 2008). In addition, prenatal glucocorticoid treatment alters dynamics in the expression of 3ß-hydroxysteroid dehydrogenase, an enzyme involved in testosterone synthesis, and decreases the expression of inhibin-α (Pedrana et al., 2016). These effects accompany a reduction in the proliferation of Leydig cells, but not Sertoli cells (Pedrana et al., 2008, 2016), as well as changes the levels of Caspase, Bcl-2, and Bax, shifting the balance from inhibition to promotion of apoptosis (Pedrana et al., 2008). Therefore, it seems likely that stress in the pregnant ewe will affect the proliferation and differentiation of cells in the developing testis of ram fetuses. Research is required to determine whether there are serious consequences for fertility in mature rams under farm conditions.

DoHaD and epigenetics

Changes in the epigenome are mediated by, for example, DNA methylation, histone modifications, and chromatin remodeling. Sinclair et al. (2016) reviewed the role of such mechanisms in DOHaD for commercial livestock, including the effects of maternal nutrition and stress during gestation, and the risks associated with advanced breeding technologies. They concluded that there is strong evidence for epigenetic processes. More recently, the field was re-reviewed by Zhu et al. (2021) who documented further evidence for environmental factors, during embryonic and fetal development, leading to the acquisition of traits caused that can be transmitted to future generations. We need to understand these processes better so we can determine whether they have a commercial impact and, if they do, devise management strategies to prevent them. On the other hand, the development of epigenome editing (see commentary by Kaiser, 2022) might present subtle alternative ways to improve livestock performance.

i) Puberty - Nutrition, growth and body composition

One way to reduce the ‘carbon footprint’ of the sheep industry is to improve the ‘methane efficiency’ of flocks by advancing the onset of puberty. Age and body mass have long been considered the dominant factors that constrain the time of puberty, with the effect of body mass mediated by adipose tissue and its hormone, leptin. However, we now know that muscle mass also plays a role (review: Rosales et al., 2018), presenting new options for understanding metabolic inputs into the control of reproduction at brain level (where puberty is ‘decided’). Importantly, it now seems likely that genetic or management strategies that increase in meat production will also improve ‘methane efficiency’. Interestingly, accelerated muscle accumulation was accompanied by and an increase in the circulating concentrations of leptin, so this adipose hormone remains central to the induction of puberty. One possibility is that pre-pubertal acceleration of muscle accumulation is accompanied by accelerated accumulation of intramuscular fat, although other factors produced by muscle might also be acting as metabolic signals to the centres in the brain that control reproduction (Rosales et al., 2018).

## Conclusion

We have come a long way in our development of CGE sheep management, but there are still many areas of research where greater effort is needed to achieve adequate industry standards. These improvements have become urgent as we try to balance the increasing demand for animal-based food with the inevitable constraints of global heating. Many of the potential solutions are research frontiers, including: i) the effects of nutrition on reproduction control systems, at all levels - brain, gonad, uterus; ii) the processes that affect the efficiency of the ram effect; iii) the potential importance of nutrition and stress on epigenetics, and thus DoHaD, for farm animal productivity. Many of the solutions to these challenges are encompassed in the theme of the *IX International Symposium on Animal Biology of Reproduction* (ISABR 2022) - ‘trends in molecular regulation of reproductive processes’ - and they present an array of exciting opportunities for young scientists.

Finally, this review focused on reproduction, but there are equally important imperatives for research on the other components of productivity (better health, nutrition and animal welfare; methane mitigation), as well as the wide variety of modern genetic tools that will help us to overcome the constraints in CGE management.
